# Multi-Target Botanical Complex Attenuates Cellular Senescence via Bidirectional P21/P53/SIRT1 Regulation: Dual Model Validation

**DOI:** 10.3390/ijms262311394

**Published:** 2025-11-25

**Authors:** Jiaqin Wu, Nanjia Dongzhu, Chengzhou Zhao, Jialin Liang, Ningbo Wang, Dengdeng Suonan, Shengnan Sun

**Affiliations:** 1 College of Pharmacy, Qinghai University, Xining 810016, China; wjq011228@163.com (J.W.); snddxp@163.com (D.S.); 2 College of Tibetan Medicine, Qinghai University, Xining 810016, China; dznjej@163.com (N.D.); 18793091535@163.com (J.L.); wnbmfs@163.com (N.W.)

**Keywords:** botanical complex, multi-target, bidirectional regulation, P21/P53/SIRT1 axis, cellular senescence, cardiomyocytes, zebrafish model, apoptosis, senotherapeutics

## Abstract

Cellular senescence is a pivotal driver of aging and age-related diseases. This study aims to systematically investigate the anti-senescence effects and molecular mechanisms of a multi-component botanical complex (SBT) using both a D-galactose-induced senescence model in H9c2 cardiomyocytes and an H_2_O_2_-induced accelerated aging model in zebrafish. The SBT complex comprises *Solms-laubachia eurycarpa*, *Bergenia purpurascens*, *Laccifer lacca*, and *Glycyrrhiza uralensis*. Results demonstrated that SBT treatment significantly enhanced cell viability (increased from 52% to 85%) and reduced senescence-associated β-galactosidase (SA-β-gal) activity (from 41.2% to 20%). At the molecular level, SBT exerted bidirectional regulation of the P21/P53/SIRT1 axis, coordinately downregulating the pro-senescence proteins P53 and P21 while upregulating the longevity-associated deacetylase SIRT1. It also modulated the balance of apoptosis-related genes by suppressing *Bax* and enhancing *Bcl-2* expression. In the zebrafish model, SBT significantly strengthened the antioxidant defense system, as indicated by increased activities of superoxide dismutase (SOD) and catalase (CAT), elevated glutathione (GSH) levels, and reduced malondialdehyde (MDA) content. These findings confirm that SBT exerts potent anti-senescence effects through bidirectional regulation of the P21/P53/SIRT1 signaling axis, enhanced antioxidant capacity, and inhibition of apoptosis, thereby providing a mechanistic foundation for the development of natural product-based interventions against aging and related diseases.

## 1. Introduction

Cellular senescence, characterized by the irreversible arrest of the cell cycle, is a fundamental hallmark of aging and a key driver of age-related functional decline and pathologies [1,2]. The molecular underpinnings of aging involve a complex network of processes, including genomic instability [3], epigenetic alterations [4], loss of proteostasis [5], and deregulated nutrient sensing [6]. Central among these are the p53 (Tumor Protein P53) and p21 (Cyclin-Dependent Kinase Inhibitor 1A) axis, and the sirtuin family of proteins, particularly SIRT1 (Sirtuin 1), which serve as pivotal regulators of the senescence program [7]. The p53 tumor suppressor protein is activated in response to diverse cellular stresses, such as DNA damage [8], and transcriptionally upregulates p21 [9]. p21, a cyclin-dependent kinase inhibitor, subsequently induces G1 phase cell cycle arrest, thereby promoting the senescent phenotype [10]. Conversely, SIRT1, an NAD^+^-dependent deacetylase [11], exerts anti-aging effects by deacetylating key substrates including p53, FOXOs (Forkhead box O transcription factors), and PGC-1α (Peroxisome proliferator-activated receptor gamma coactivator 1-alpha) [12]. This deacetylation modulates cellular stress resistance [13], metabolic homeostasis, and inflammatory responses [14]. Notably, SIRT1-mediated deacetylation directly inhibits p53’s transcriptional activity [15], forming a delicate bidirectional regulatory circuit that finely balances cellular senescence and survival [16].

Despite significant advances in understanding aging biology, the current therapeutic arsenal for intervening in the aging process remains limited [17,18]. Pharmacological agents like rapamycin, an mTOR inhibitor, have demonstrated lifespan extension in model organisms but are often hampered by side effects and challenges in clinical translation [19]. In this context, natural products present promising alternatives due to their inherent multi-target activities and favorable safety profiles [20]. Botanical preparations from unique ecosystems, such as high-altitude regions, have attracted particular interest for their distinctive phytochemical compositions and traditional ethnopharmacological uses [21]. A notable example is Srolo Bzhtang (SBT), a defined multi-herbal Tibetan formulation composed of *Solms-laubachia eurycarpa*, *Bergenia purpurascens*, *Laccifer lacca*, and *Glycyrrhiza uralensis*. Previous phytochemical studies have identified a complex mixture of bioactive constituents in SBT, including flavonoids (e.g., liquiritin and isoliquiritin from *Glycyrrhiza uralensis*), phenolic acids (e.g., bergenin from *Bergenia purpurascens*), tannins, and unique polysaccharides, which collectively contribute to its observed pharmacological effects [22]. Prior pharmacological investigations have confirmed SBT’s anti-inflammatory and antioxidant properties, primarily attributed to its ability to scavenge reactive oxygen species (ROS) and inhibit inflammatory cytokine signaling [23,24,25]. Building on this foundation, recent studies have further elucidated its efficacy in ameliorating pulmonary hypertension and attenuating airway inflammation [26,27,28]. However, its potential to directly counteract cellular aging, particularly within cardiac contexts and through modulation of the core P21/P53/SIRT1 senescence axis, remains largely unexplored and represents a significant gap in knowledge.

This study was therefore designed to systematically investigate the anti-senescence capacity and underlying molecular mechanisms of SBT. Utilizing a dual-model validation approach—comprising a D-galactose-induced senescence model in H9c2 cardiomyocytes and an H_2_O_2_-induced accelerated aging model in zebrafish [29,30]—we comprehensively evaluated the extract’s efficacy in mitigating senescence-associated phenotypes, oxidative stress parameters, and key molecular alterations within the P21/P53/SIRT1 signaling axis. Our integrated findings not only illuminate the novel anti-aging mechanisms of SBT but also position it as a compelling multi-target botanical candidate for development as a serotherapeutic intervention against age-related degenerative diseases.

## 2. Results

### 2.1. SBT Treatment Mitigates D-Galactose-Induced Oxidative Stress in H9c2 Cardiomyocytes

**Figure 1:**
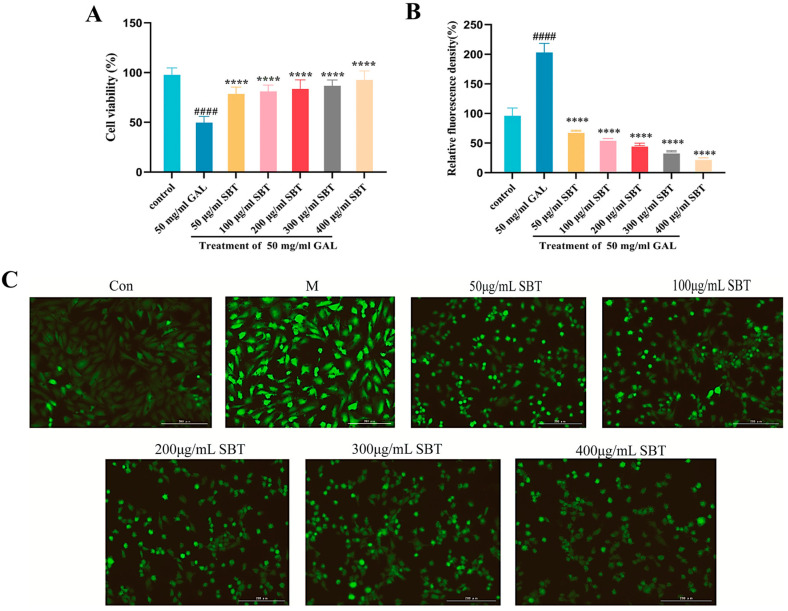
SBT alleviates D-galactose-induced oxidative damage in H9c2 cells. (**A**) CCK-8 assay assessment of cellular viability. (**B**) Quantitative analysis of intracellular ROS levels by fluorescence. (**C**) Representative micrographs of ROS fluorescence (magnification 20×, scale bar = 200 μm). #### *p* < 0.0001 versus the Control group; **** *p* < 0.0001 versus the GAL group.

The protective effects of SBT against D-galactose (GAL)-induced oxidative damage in H9c2 cardiomyocytes were evaluated by measuring cell viability using the CCK-8 assay and intracellular ROS levels via fluorescence staining. Exposure to GAL significantly reduced cell viability to 52% of the control level (set as 100%). In contrast, SBT intervention markedly restored the survival rate to 85 ± 5.2% (*p* < 0.0001; Figure 1A). Fluorescence imaging revealed substantial ROS accumulation in the GAL-treated group, which was significantly attenuated by SBT treatment, indicating its potent antioxidant capacity (Figure 1C). These results demonstrate that SBT effectively alleviates GAL-induced cardiomyocyte injury through its antioxidant activity.

### 2.2. SBT Treatment Attenuates D-Galactose-Induced Cellular Senescence in H9c2 Cardiomyocytes

**Figure 2:**
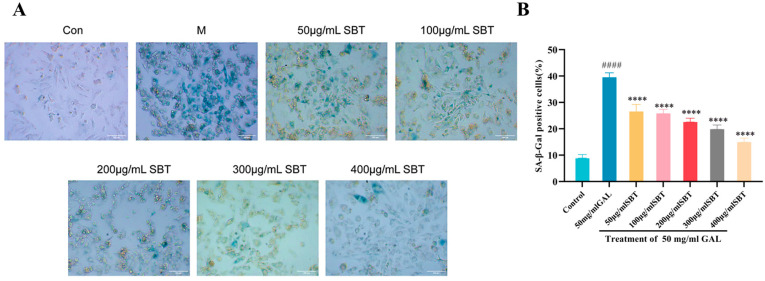
SBT attenuates D-galactose-induced cellular senescence in H9c2 cardiomyocytes. (**A**) Representative micrographs of SA-β-gal staining (magnification 20×, scale bar = 200 μm). (**B**) Quantitative analysis of the proportion of SA-β-gal-positive cells. #### *p* < 0.0001 versus the Control group; **** *p* < 0.0001 versus the GAL group.

To investigate the anti-senescence effect of SBT, H9c2 cells were treated with GAL to establish a senescence model, which was assessed by SA-β-gal staining. The model group exhibited a significant increase in senescence burden (41.2% vs. control, *p* < 0.0001). In contrast, SBT treatment substantially reversed this effect, reducing the proportion of SA-β-gal-positive cells to 20 ± 5.0% (*p* < 0.0001; Figure 2). These results indicate that SBT effectively eliminates GAL-induced senescent cells.

### 2.3. SBT Alleviates D-Galactose-Induced Apoptosis in H9c2 Cardiomyocytes

**Figure 3:**
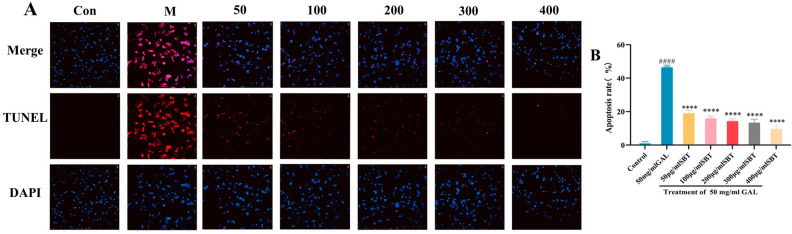
SBT inhibits apoptosis in H9c2 cells caused by D-galactose. (**A**) TUNEL staining (magnification 10×, scale bar = 100 μm). (**B**) Quantitative analysis of TUNEL-positive cells. #### *p* < 0.0001 versus the Control group; **** *p* < 0.0001 versus the GAL group.

To determine if SBT could inhibit programmed cell death in senescent H9c2 cells, we employed TUNEL staining. The results revealed that SBT administration markedly decreased the apoptotic rate compared to the GAL group (Figure 3A), underscoring its anti-apoptotic efficacy. In summary, these results indicate that SBT reduces apoptosis in H9c2 cardiomyocytes triggered by D-galactose.

### 2.4. SBT Modulates the P53/P21/SIRT1 Pathway in D-Galactose-Induced H9c2 Senescent Cells

**Figure 4:**
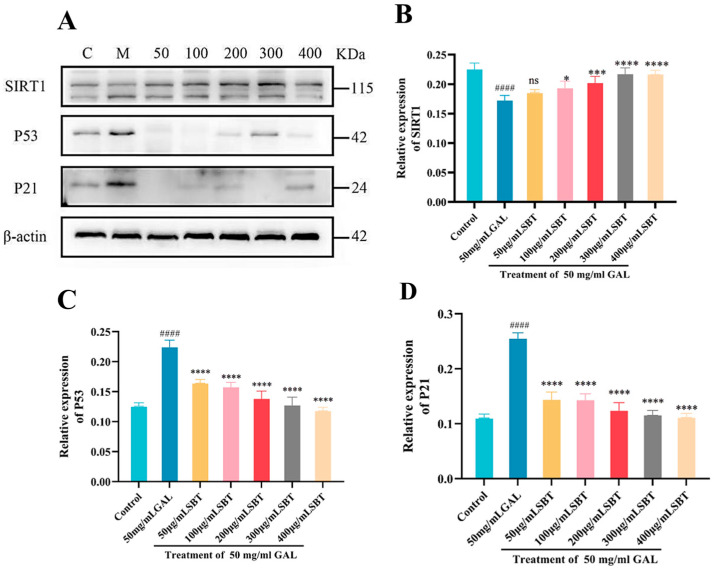
SBT regulates senescence-associated proteins in H9c2 cells. (**A**) Western blot analysis of P53, P21, and SIRT1 expression. (**B**–**D**) Quantitative analysis of (**B**) P53, (**C**) P21, and (**D**) SIRT1. #### *p* < 0.0001 versus Control; **** *p* < 0.0001, *** *p* < 0.001, * *p* < 0.05 versus GAL group; ns, not significant.

The P21/P53/SIRT1 axis serves as a core regulatory network governing senescence, integrating signals that modulate cell cycle progression, DNA damage response, and oxidative stress homeostasis. To explore SBT’s impact on this pathway, Western blotting quantified P21, P53, and SIRT1 protein levels. Our data showed that SBT treatment led to the downregulation of the pro-senescence proteins P21 and P53 (Figure 4A). Conversely, there was a marked increase in SIRT1 expression (Figure 4A), implicating the activation of this deacetylase in the cardioprotective mechanism of SBT. In summary, these results indicate that SBT modulates the P53/P21/SIRT1 pathway in H9c2 cardiomyocytes, suggesting a mechanism by which it may alleviate cellular senescence.

### 2.5. SBT Improves the Survival and Cardiac Function of H_2_O_2_-Treated Zebrafish

**Figure 5:**
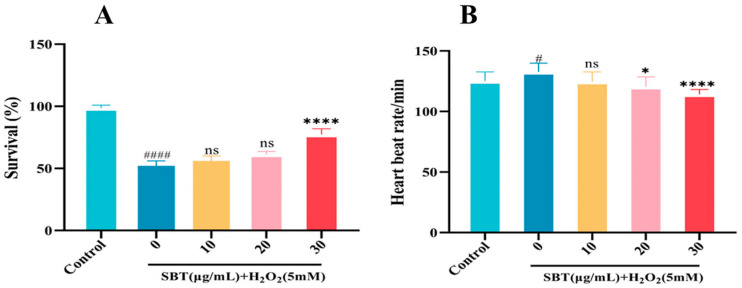
SBT exerts protective effects on survival and heart rate in H_2_O_2_-induced zebrafish model. (**A**) Survival rate analysis. (**B**) Quantitative assessment of heart rate. # *p* < 0.05, #### *p* < 0.0001 versus Control; **** *p* < 0.0001, * *p* < 0.05 versus H_2_O_2_ group; ns, not significant.

We further investigated the protective effects of SBT in an H_2_O_2_-induced zebrafish aging model. As illustrated in Figure 5A,B, SBT treatment significantly improved the survival rate and restored the heart rate of zebrafish larvae impaired by H_2_O_2_ exposure. Together, these results demonstrate that SBT effectively improves overall viability and cardiac function in a zebrafish model of accelerated aging.

### 2.6. SBT Enhances the Antioxidant Defense in H_2_O_2_-Treated Zebrafish

**Figure 6:**
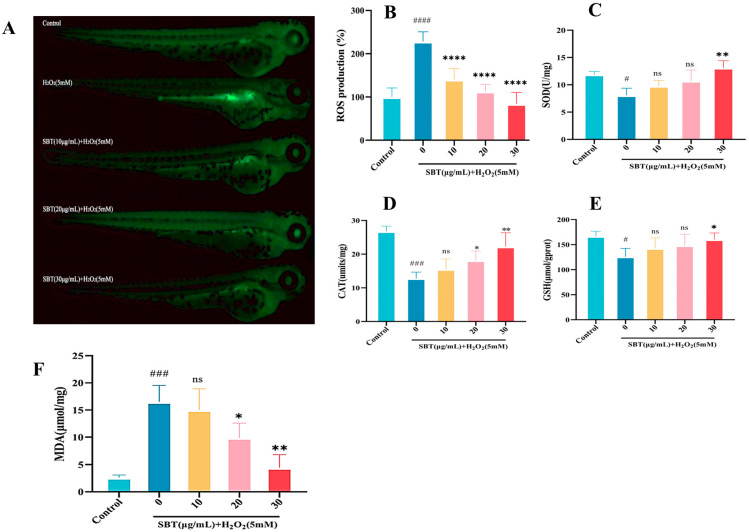
SBT alleviates oxidative stress in H_2_O_2_-treated zebrafish larvae. (**A**) ROS staining. (**B**) Quantification of relative ROS fluorescence intensity. (**C**) SOD activity. (**D**) CAT activity. (**E**) GSH level. (**F**) MDA content. # *p* < 0.05, ### *p* < 0.001, #### *p* < 0.0001 versus Control; **** *p* < 0.0001, ** *p* < 0.01, * *p* < 0.05 versus H_2_O_2_ group; ns, not significant.

We further investigated the protective effects of SBT in an H_2_O_2_-induced zebrafish aging model by assessing its antioxidant capacity. Fluorescence-based ROS detection showed that the pronounced oxidative stress induced by H_2_O_2_ was markedly mitigated by SBT intervention (Figure 6A,B). Antioxidant enzyme activity and metabolite levels were additionally quantified. The administration of SBT improved the redox status, as evidenced by increased activities of SOD (Figure 6C) and CAT (Figure 6D), elevated GSH levels (Figure 6E), and reduced MDA content (Figure 6F). Together, these results demonstrate that SBT enhances the antioxidant defense system in a zebrafish model of accelerated aging.

### 2.7. SBT Inhibits Aging and Cell Death in Hydrogen Peroxide-Exposed Zebrafish Embryos

**Figure 7:**
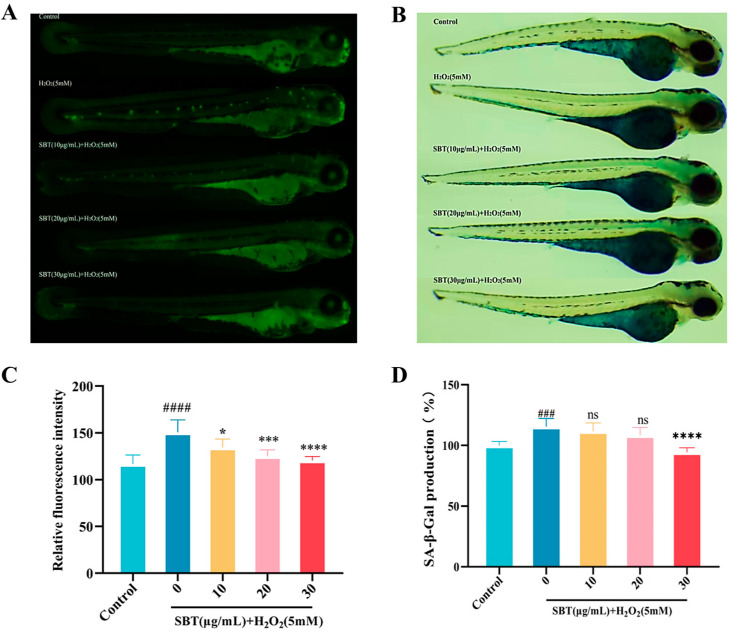
SBT mitigates H_2_O_2_-induced apoptosis and cellular senescence in zebrafish larvae. (**A**) AO staining. (**B**) SA-β-gal staining. (**C**) AO fluorescence intensity. (**D**) Quantitative analysis of β-galactosidase staining. ### *p* < 0.001, #### *p* < 0.0001 versus Control group; **** *p* < 0.0001, *** *p* < 0.001, * *p* < 0.05 versus H_2_O_2_ group; ns, not significant.

To investigate the potential of SBT in mitigating programmed cell death and delaying age-related decline in senescent zebrafish, acridine orange (AO) staining was performed. A significant increase in apoptotic signals (AO-positive) appeared in the heart tissue of zebrafish in the model cohort. In contrast, SBT intervention markedly reduced AO-positive signals (Figure 7A). Furthermore, the SA-β-gal assay demonstrated a marked escalation of senescence-linked enzymatic responses in the model cohort, an effect that was effectively reversed by SBT treatment (Figure 7B).

### 2.8. SBT Effects on P53/P21/SIRT1 Signaling Pathway

**Figure 8:**
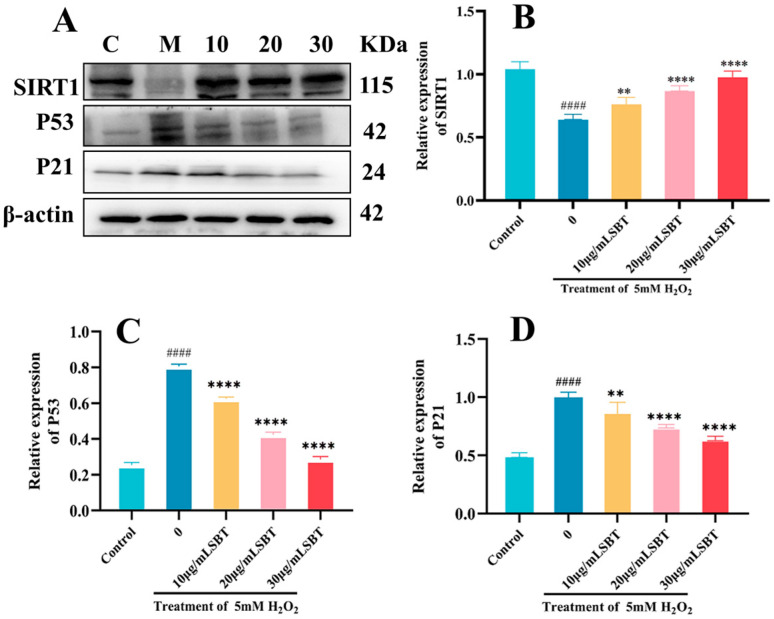
SBT modulates the P53/P21/SIRT1 pathway in zebrafish. (**A**) Representative Western blots of P21, P53, and SIRT1. (**B**–**D**) Quantifying SIRT1, P53, and P21 protein levels. #### *p* < 0.0001 versus Control group; **** *p* < 0.0001, ** *p* < 0.01 versus H_2_O_2_ group.

Western blot analysis assessed key protein expression in the SBT-modified P21/P53/SIRT1 signaling pathway. Figure 8A illustrates that SBT therapy markedly decreased P53 and P21 protein levels while concurrently increasing SIRT1 expression.

### 2.9. SBT-Mediated Modulation of Senescence-Associated Gene Transcription

**Figure 9:**
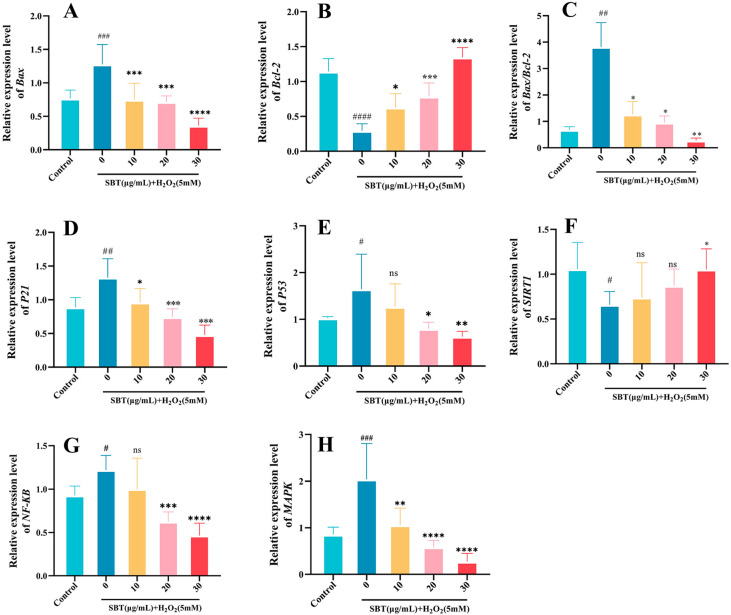
Effects of SBT on senescence-related gene expression in zebrafish. (**A**–**H**) Quantification of mRNA for *Bax*, *Bcl-2*, *P21*, *P53*, *SIRT1*, *NF-κB*, and *MAPK*, and the *Bax/Bcl-2* ratio. ### *p* < 0.001, ## *p* < 0.01, #### *p* < 0.0001, # *p* < 0.05 versus control group; **** *p* < 0.0001, *** *p* < 0.001, ** *p* < 0.01, * *p* < 0.05 versus H_2_O_2_ group; ns, not significant.

Quantitative PCR (q-PCR) assessed SBT’s transcriptional impacts through mRNA level measurements of key genes regulating apoptosis (*Bax*, *Bcl-2*), inflammation (*NF-κB*, *MAPK*), and senescence (*P21*, *P53*, *SIRT1*). The results demonstrated that SBT administration significantly suppressed *Bax* transcript levels while enhancing *Bcl-2* expression (Figure 9A,B), resulting in a reduced *Bax/Bcl-2* ratio (Figure 9C). A concurrent decrease was observed in *P21* and *P53* mRNA levels (Figure 9D,E), whereas *SIRT1* expression was notably elevated (Figure 9F). Furthermore, *NF-κB* and *MAPK* signaling components showed marked transcriptional downregulation relative to the H_2_O_2_ group (Figure 9G,H). The collective data indicate that coordinated modulation of gene expression is the mechanism through which SBT attenuates cellular senescence.

## 3. Discussion

Our study provides compelling evidence that the botanical formulation Srolo Bzhtang (SBT) confers robust anti-senescence effects by orchestrating a multi-targeted response centered on the P21/P53/SIRT1 signaling axis [31]. Consistent findings in D-galactose-induced senescent H9c2 cardiomyocytes and H_2_O_2_-accelerated zebrafish aging models underscore the therapeutic potential of SBT in mitigating cellular aging and its associated functional decline [32].

A pivotal finding of this work is the elucidation of SBT’s ability to coordinately modulate the P53/P21/SIRT1 network, a crucial hub integrating stress responses with cellular fate decisions [33]. In senescent H9c2 cells, SBT not only rescued cell viability but also fundamentally altered the expression landscape of key senescence regulators, suppressing the pro-senescence proteins P53 and P21 while promoting the expression of the longevity-associated deacetylase SIRT1 [34]. The recapitulation of these molecular changes-downregulation of P53/P21 and upregulation of SIRT1-in the H_2_O_2_-induced zebrafish model strongly implies a conserved mechanism of action across species [35]. This coordinated regulation suggests a plausible model wherein SBT-mediated activation of SIRT1 leads to the deacetylation and functional inhibition of P53 [36], thereby attenuating the transcription of its downstream target P21 and subsequently alleviating cell cycle arrest driven by P21 and the establishment of the senescent phenotype [37,38].

First, our results clearly demonstrate that SBT exerts its anti-senescence effects through potent antioxidant activity. Beyond its core action on the senescence axis, SBT’s efficacy is deeply rooted in its potent capacity to reinforce the cellular defense system against oxidative stress [39]. In zebrafish, SBT treatment significantly bolstered the endogenous antioxidant arsenal, enhancing the activities of SOD and CAT and elevating GSH levels [40]. The concomitant reduction in MDA content indicates successful attenuation of lipid peroxidation [41]. This antioxidant capacity was corroborated in the H9c2 cell model, where SBT significantly reversed D-galactose-induced intracellular ROS accumulation (Figure 1), mirroring its in vivo effects in zebrafish. This comprehensive enhancement of the cellular antioxidant defenses effectively neutralizes excessive ROS accumulation, a key driver of aging, thereby preserving cellular integrity from oxidative insult [42,43].

Second, the anti-senescence profile of SBT is further augmented by its anti-apoptotic properties [44]. Our data demonstrate that SBT shifts the apoptotic balance towards cell survival, as evidenced by the downregulation of the pro-apoptotic gene *Bax* and the upregulation of the anti-apoptotic gene *Bcl-2* [45]. This conclusion was directly supported by TUNEL staining in H9c2 cells (Figure 3) and AO staining in zebrafish (Figure 7A,C), showing that SBT markedly reduced apoptosis in the senescent milieu. qPCR results further confirmed the regulatory effect of SBT on the *Bax/Bcl-2* ratio at the transcriptional level (Figure 9A–C), clarifying the molecular basis for its anti-apoptotic action.

Furthermore, our study revealed that SBT modulates senescence-associated inflammatory signaling, as evidenced by the downregulation of critical elements within the MAPK/NF-κB pathways, indicating a concomitant dampening of inflammatory and stress responses [46]. qPCR data showed that SBT treatment significantly reduced the mRNA levels of *NF-κB* and *MAPK* (Figure 9G,H), suggesting that SBT may further ameliorate the senescent microenvironment by suppressing SASP-associated inflammatory signaling. This multi-faceted intervention, targeting oxidative damage, programmed cell death, and associated inflammatory signaling, establishes an integrated defensive network against the drivers of cellular aging [47].

The experimental paradigm employed, leveraging the complementary strengths of the H9c2 cell line and the zebrafish model, provided a powerful platform for validation [48]. This dual-model system enabled a multi-tiered and comprehensive validation, spanning from cellular viability, oxidative stress, and senescence markers (SA-β-gal) to apoptosis, key signaling pathways (P21/P53/SIRT1, MAPK/NF-κB), and overall physiological functions (zebrafish survival and heart rate), thereby greatly enhancing the reliability of our conclusions [49,50]. Based on these findings, future investigations should prioritize several promising directions. First, employing genetic (e.g., siRNA) or pharmacological inhibition of SIRT1 is crucial to definitively establish a causal relationship between SIRT1 activation and the observed P53 deacetylation and subsequent anti-senescence effects. Second, a detailed pharmacokinetic profiling of SBT’s bioactive constituents is essential to understand their in vivo bioavailability and metabolism. Furthermore, deconvoluting the synergistic interactions among the multiple active components within the SBT formulation will be key to appreciating the holistic nature of its polypharmacological action.

In conclusion, this study delineates a novel mechanism by which SBT alleviates cellular senescence primarily through the coordinated targeting of the P21/P53/SIRT1 signaling axis. By reinforcing antioxidant defenses, inhibiting apoptosis, and modulating associated inflammatory signals, SBT effectively clears senescent cells and ameliorates aging-associated phenotypes. By enhancing the clearance of senescent cells and ameliorating aging-associated phenotypes through reinforced antioxidant defenses and suppressed apoptosis, SBT emerges as a promising, multi-targeted candidate for further development as a therapeutic or preventive agent for age-related degenerative conditions.

## 4. Materials and Methods

Srolo Bzhtang (abbreviated as SBT) was provided by the Pharmaceutical Preparation Department of Qinghai Tibetan Medical Hospital (Xining, China) (Batch No. 20130607). D-galactose was purchased from Sangon Biotech (Shanghai, China) Co., Ltd. (Shanghai, China) (Catalog Number A600215-0500).

### 4.1. Cell Culture and Drug Treatment

H9c2 rat cardiomyocytes obtained from the Cellular Repository of Peking Union Medical College (Beijing, China). Cells were maintained in high-glucose DMEM (BBI, Shanghai, China; Cat# E600003-0500) containing 10% fetal bovine serum (FBI, Catalog Number E600001-0500) and 1% penicillin/streptomycin (BBI, Catalog Number E607011-0100), Cultivated at 37 °C with 5% CO_2_ and humid conditions. Subculturing was performed with 0.25% trypsin-EDTA (BBI, Catalog Number E607002-0100) upon reaching 80–90% confluency. All cultures routinely undergo mycoplasma testing to ensure the absence of contamination. To induce cellular senescence, cells were subjected to 50 mg/mL D-galactose treatment for 24 h; control cells received an equal volume of complete medium. Following induction, the samples were categorized into the subsequent experimental subsets: normal control (untreated), model (GAL group; treated with 50 mg/mL D-galactose for 24 h), SBT-treated (exposed to D-galactose followed by 24 h incubation with SBT at 50, 100, 200, 300, or 400 μg/mL).

### 4.2. Cell Viability Assay H9c2

H9c2 cells seeded at 5 × 10^3^ cells/well in 96-well plates underwent 24-h incubation to facilitate cell attachment. After medium depletion, the cells were allocated into groups: the model and treatment groups were both exposed to 50 mg/mL D-galactose (GAL), in contrast to the control group, which was replenished with fresh complete medium. At the 24-h mark, the supernatant was aspirated. The treatment groups were then administered varying concentrations of SBT (50, 100, 200, 300, or 400 μg/mL), with the control and model groups receiving an equivalent volume of complete medium. Incubation proceeded for a further 24 h, at which point CCK-8 reagent (BBI, Catalog Number E606335-2000) was added to each well. The plate was incubated at 37 °C with 5% CO_2_ for 30 min. The absorption was measured at 450 nm via a microplate reader.

### 4.3. ROS Staining

ROS levels were assessed with the Beyotime DCFH-DA fluorescent dye (Shanghai, China; Catalog Number S0033S), following the supplier’s instructions. Briefly, H9c2 cells grown in 24-well plates were washed and incubated in a 10 μM DCFH-DA solution in serum-free conditions at 37 °C for a duration of 30 min. To eliminate residual dye, three washes with serum-free medium were performed post-staining. For the zebrafish larvae, the procedure was analogous: they were placed in a 10 μM DCFH-DA solution prepared in embryo medium and incubated at 37 °C for 30 min. Thereafter, the larvae underwent three 5-min washes in embryo medium to thoroughly clear any unincorporated probe. A confocal laser scanning microscope (ZEISS LSM 900, Oberkochen, Germany) was employed to capture fluorescence images. Finally, the relative ROS levels were quantified by analyzing the fluorescence intensity with ImageJ software (v1.48v, National Institutes of Health, Bethesda, MD, USA).

### 4.4. SA-β-Gal Staining

According to the manufacturer’s protocol, cellular senescence was evaluated by detecting SA-β-gal enzymatic function via a commercial assay (Beyotime, Catalog Number C0602). In brief, cells in 6-well plates were fixed for 10 min at room temperature, subjected to two PBS washes, and then subjected to an incubation period of 12 h in a stain solution at 37 °C. The percentage of cells positive for SA-β-gal was quantified by examination under a light microscope. For the zebrafish larvae, the procedure involved three rinses with embryo medium prior to fixation at 4 °C overnight. Following fixation, after triple washing with embryo medium, the larvae were incubated overnight at 37 °C in staining solution. Zebrafish SA-β-gal activity was captured via MZX100 stereomicroscope (Mshot, Guangzhou, China).

### 4.5. TUNEL Staining

Assessment of cell apoptosis was conducted using a TUNEL kit (Beyotime, Catalog Number C1086) from a commercial supplier as per the supplied instructions. The procedure commenced with the fixation of cells cultured in 6-well plates, using 4% paraformaldehyde treatment for 30 min at ambient temperature. Subsequently, a permeabilization step was performed to allow the assay reagents to penetrate the cells. Following this, the specimens underwent 60 min of dark incubation at 37 °C using the TUNEL reagent. Imaging was conducted on a ZEISS LSM 900 confocal microscope, and apoptotic cells were identified by the presence of red fluorescent nuclei in the acquired micrographs.

### 4.6. Western Blotting

RIPA buffer (Beyotime, Catalog Number P0013B) isolated proteins from collected cell cultures and zebrafish tissues. Following quantification of protein concentration with a BCA assay kit (Thermo Scientific, Waltham, MA, USA; Catalog Number 23225), Samples were thermally denatured in loading buffer at 100 °C for 10 min. Subsequently, the proteins underwent electrophoretic separation on 10% SDS-PAGE gels and were electrophoretically transferred to PVDF membranes using a wet transfer system. Membranes were incubated in 5% skim milk, followed by overnight primary antibody application at 4 °C. The primary antibodies used and their dilutions were: P21 (Proteintech, Chicago, IL, USA; Catalog Number 10355-1-AP; 1:1000), P53 (Proteintech, Catalog Number 100442-1-AP; 1:1000), and SIRT1 (Proteintech, Catalog Number 13161-1-AP; 1:1000). Following three TBST washes, the membranes underwent a 2-h incubation period at ambient temperature with HRP-conjugated secondary antibodies, specifically goat anti-rabbit IgG (BBI, Catalog Number D110058; dilution 1:5000) and goat anti-mouse IgG (BBI, Catalog Number D110087; dilution 1:5000). Signal development was achieved with an ECL chemiluminescence substrate (Millipore, Burlington, MA, USA; Product CodeWBKLS050), The band intensities were measured through grayscale analysis with ImageJ software (v1.48v, National Institutes of Health, Bethesda, MD, USA), utilizing β-actin (Proteintech, Catalog Number 8111S-1-RR; 1:5000) as a loading control.

### 4.7. Maintenance and Experimental Manipulation of Zebrafish

Zebrafish (AB wild-type strain) were obtained from the China Zebrafish Resource Center (Wuhan, China). Embryos were gathered through natural spawning, with a ratio of females to males of 1:2. For further experiments, normally developed embryos at 8 h post-fertilization (hpf) were chosen. Roughly 25 zebrafish embryos at 8 hpf were placed in 12-well plates filled with embryo medium and co-incubated in embryo water enriched with 5 mM H_2_O_2_ until they reached 24 hpf. After substituting with fresh embryo water, the embryos were maintained for an additional 2 days to create the H_2_O_2_-induced senescence model. The zebrafish embryos were subsequently divided into several groups: the control group (untreated); the hydrogen peroxide (H_2_O_2_) group (exposed to embryo water containing 5 mM H_2_O_2_ until 24 hpf); the SBT treatment group (embryos first received embryo water with 10, 20, or 30 μg/mL SBT for 1 h before adding embryo water with 5 mM H_2_O_2_, continuing co-incubation until 24 hpf).

### 4.8. Survival Rate and Heartbeat Rate/Time Analysis

At 8 h post-fertilization (hpf), zebrafish embryos were individually transferred into 12-well plates, with approximately 25 embryos per group. They were then exposed to embryo medium containing SBT at concentrations of 10, 20, or 30 μg/mL for 1 h. Following this pretreatment, the embryos were challenged with 5 mM H_2_O_2_ in embryo medium until 24 hpf. After the medium was exchanged for fresh embryo medium, the embryos were maintained for a further 48 h. Survival rates were determined by counting viable larvae, while heart rates were quantified for the surviving individuals under a stereomicroscope. Only viable larvae were subsequently used for further experiments.

### 4.9. Measurement of Antioxidant Capacity

The antioxidant capacity of zebrafish larvae was evaluated using commercial assay kits according to the manufacturers’ instructions. Specifically, superoxide dismutase (SOD) activity and malondialdehyde (MDA) concentration (an indicator of lipid peroxidation) were measured using the SOD activity assay kit (Beyotime, Catalog Number S0101) and MDA quantitative kit (Beyotime, Catalog Number S0131), respectively. Glutathione assay kits measured decreased GSH concentrations (Beyotime, Catalog Number S0052), while CAT activity was examined using a catalase-specific detection system (Beyotime, Catalog Number S0051). All absorbance measurements were conducted by following the standardized protocols provided in the respective kit manuals.

### 4.10. Acridine Orange (AO) Staining

Zebrafish larvae from each experimental group were incubated in embryo medium containing acridine orange (AO) staining solution (Sigma-Aldrich, St. Louis, MO, USA; Product Code A6014) at 37 °C for 20 min. Following incubation, the larvae underwent three washes in fresh embryo medium. Imaging by fluorescence was conducted with a stereo microscope fitted with the appropriate filters, and the acquired images were quantitatively analyzed for fluorescence intensity using ImageJ software (v1.48v, National Institutes of Health, Bethesda, MD, USA).

### 4.11. qRT-PCR

Following isolation from zebrafish larvae using TRIzol reagent (Thermo Fisher Scientific, Waltham, MA, USA; Catalog Number 15596026), RNA was converted to cDNA using PrimeScript RT reagent (Takara Bio, Kusatsu, Japan; Catalog Number RR047A). qPCR analysis was subsequently conducted with the aid of TB Green Premix Ex Taq II (Tli RNaseH Plus; Takara Bio, Catalog Number RR820A). All experimental steps were rigorously performed according to the manufacturers’ protocols to ensure reproducibility. Data normalization was achieved using β-actin as an endogenous control. Table 1 lists all primer sequences employed in the present study.

### 4.12. Statistical Analysis

Statistical processing was utilized with GraphPad Prism 9 software. Quantitative datasets were initially assessed for normality. Data that followed a normal distribution are presented as the mean ± standard deviation. Statistical comparisons between groups were performed using Student’s *t*-test or one-way ANOVA, with a *p*-value of less than 0.05 considered statistically significant.

## Figures and Tables

**Table 1:** Primer sequences used in this study.

Gene Name	Forward Primer (5′-3′)	Reverse Primer (5′-3′)
*Bax*	CGGCATGGCGACAGGGATG	CATAGCAGGAGACGGTGGTGATG
*Bcl-2*	CTCCTTCTCATACTTCAGCCTCCAC	ACCTTCAATGCCTCCTCCATCTTAC
*MAPK*	GTCCTACAGCAGCACAACTTCTAC	TCAACCCACAACGAAACACTCAG
*NF-κB*	CCTGTCTGTCTGTCTGTCTGTCTG	TCGTGGTGTCGTTGCTCTTCTC
*p21*	CCAGAGACGACACCGTTTATT	GGAAGACTGAGGAATGGATCTTT
*p53*	CGAGCCACTGCCATCTATAA	CTGATTGCCCTCCACTCTTATC
*SIRT1*	CGCAAAGACATCAACACGTTAG	CAGGAATCCCACAGGAAACA
*β-actin*	TCGAGCAGGAGATGGGAACC	CTCGTGGATACCGCAAGATTC

## Data Availability

The original contributions presented in the study are included in the article. Further inquiries can be directed to the corresponding author.

## Abbreviations

SBT	Srolo Bzhtang
GAL	D-galactose
ROS	Reactive Oxygen Species
SA-β-gal	Senescence-Associated β-galactosidase
SOD	Superoxide Dismutase
CAT	Catalase
GSH	Glutathione
MDA	Malondialdehyde
AO	Acridine Orange
MAPK	Mitogen-Activated Protein Kinase
NF-κB	Nuclear Factor kappa-light-chain-enhancer of activated B cells
Bax	Bcl-2-Associated X protein
Bcl-2	B-cell lymphoma 2
SIRT1	Silent Information Regulator 1
DMEM	Dulbecco’s Modified Eagle Medium
FBS	Fetal Bovine Serum
CCK-8	Cell Counting Kit-8
DCFH-DA	2′,7′-Dichlorodihydrofluorescein diacetate
RIPA	Radioimmunoprecipitation Assay
SDS-PAGE	Sodium Dodecyl Sulfate-Polyacrylamide Gel Electrophoresis
PVDF	Polyvinylidene Fluoride
HRP	Horseradish Peroxidase
ECL	Enhanced Chemiluminescence
hpf	Hours Post-Fertilization
TBST	Tris-Buffered Saline with Tween-20
ANOVA	Analysis of Variance
